# Use of antivenoms for the treatment of envenomation by Elapidae snakes in Guinea, Sub-Saharan Africa

**DOI:** 10.1186/1678-9199-19-6

**Published:** 2013-03-28

**Authors:** Mamadou C Baldé, Jean-Philippe Chippaux, Mamadou Y Boiro, Roberto P Stock, Achille Massougbodji

**Affiliations:** 1 Institut Pasteur de Guinée, Kindia, Guinée; 2 Institut de Recherche pour le Développement, Cotonou, Bénin; 3 Centre d’Etude et de Recherche sur le Paludisme Associé à la Grossesse et à l’Enfance, Cotonou, Bénin; 4 Instituto de Biotecnología, Universidad Nacional Autónoma de México, Cuernavaca, México

**Keywords:** Elapid, Neurotoxins, Treatment, Antivenom, Guinea, Africa

## Abstract

**Background:**

In Guinea Elapids are responsible for 20% of envenomations. The associated case fatality rate (CFR) ranged 15-27%, irrespective of treatment.

**Results:**

We studied 77 neurotoxic envenomations divided in 3 groups: a set of patients that received only traditional or symptomatic treatments, and two other groups that received either 2 or 4 initial vials of Antivipmyn® Africa renewed as necessary. CFR was 27.3%, 15.4% and 17.6%, respectively. Although antivenom treatment was likely to reduce CFR, it didn’t seem to have an obvious clinical benefit for the patients, suggesting a low treatment efficacy. Mean delay to treatment or clinical stages were not significantly different between the patients who recovered and the patients who died, or between groups. Interpretation of these results is complicated by the lack of systematic studies under comparable conditions. Of particular importance is the absence of assisted ventilation, available to patients in all the other clinical studies of neurotoxic envenomation.

**Conclusion:**

The apparent lack of clinical benefit may have several causes. The hypothesis of a limited therapeutic window, i.e. an insufficient formation of antigen-antibody complexes once toxins are bound to their targets and/or distributed beyond the reach of antivenom, should be explored.

## Background

The efficacy of immunotherapy and its role in the treatment of envenomation are well established and not in question, at least in Africa [1,2]. The limited availability of antivenoms has led several manufacturers from emergent countries to propose their services to alleviate this critical deficit [3-6]. Two clinical studies conducted in northern Cameroon in 1993 and 1996 established the safety of F(ab’)_2_-based antivenoms administered by perfusion or direct intravenous injection [7,8]. Between 2005 and 2006, a clinical study using Antivipmyn® Africa, with results judged to be very successful, was conducted in Benin [9]. However, these clinical studies were concerned essentially with envenomations caused by Viperidae, particularly *Echis ocellatus*, a species largely predominant in the savannas of West Africa. A clinical study in Guinea confirmed the apparent general efficacy and safety of Antivipmyn® Africa [10]. However, it revealed shortcomings regarding the treatment of envenomations by Elapidae.

**Figure 1:**
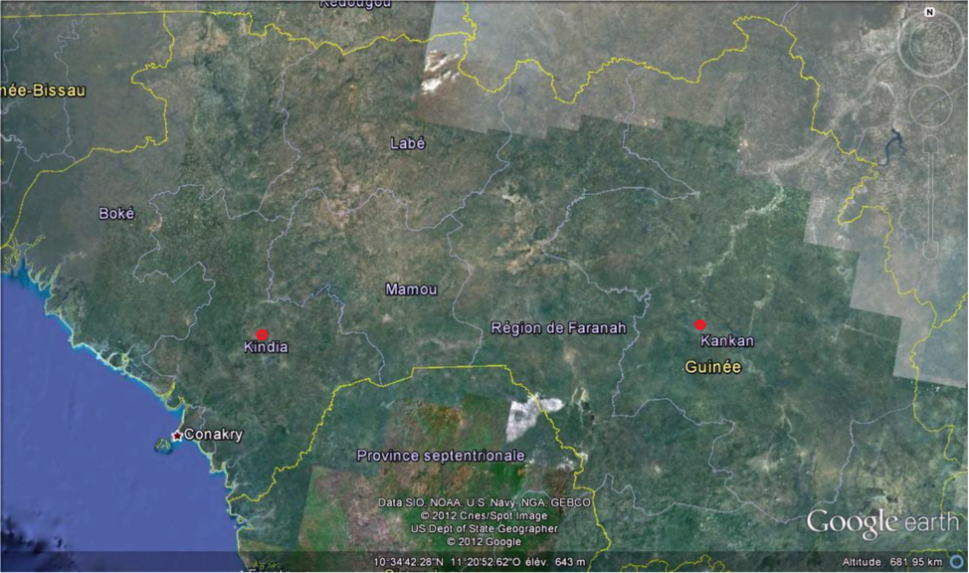
Location of survey sites in Guinea.

We have reexamined the available records in Upper and Lower Guinea (Figure 1) to evaluate the efficacy and shortcomings of immunotherapy relative to the absence of specific treatment in patients with overt neurological symptoms due to envenomation by African Elapidae. Patients were divided into three groups according to treatment protocol.

## Methods

An initial group of 33 patients was retrospectively composed after assemblage of clinical information from hospitalization registries of a health center in the region of Kankan (Upper Guinea). None of them had received antivenom as it was unavailable at that time. Two other groups were based on prospective clinical studies at the Pasteur Institute of Guinea in Kindia (Lower Guinea) in order to ascertain the safety and efficacy of Antivipmyn® Africa under real field conditions, the results of which have been published elsewhere [10]. The second group included 26 patients who were treated during the years 2009 and 2010. Patients received an initial dose of 2 vials of antivenom renewed as a function of clinical evolution 3, 6, 12 and 24 hours after the first dose (low-dose group). Since the results appeared unsatisfactory, the design of the study was amended in 2011 to increase the dose of antivenom accordingly. The third group included 18 patients (but only 17 treated because one died on arrival before treatment). They received twice the initial dose (4 vials), renewed as a function of evolution 3, 6, 12 and 24 hours after the initial dose (high-dose group).

We included all bitten patients who had presented neurological disorders: local paresthesia, tremors and muscular contractions, palpebral ptosis, vision impairment, tinnitus, dysarthria, consciousness disorders, dyspnea, hypersecretion. The severity and surveillance of envenomation were evaluated by means of scores (Table 1) [10].

**Table 1:** Gradation of neurological symptoms

**Score**	**Symptoms**
**Grade 1**	Local paraesthesia (anesthesia, tingling, stinging)
**Grade 2**	Hypersecretion (sweat, saliva)
**Grade 3**	Impaired vision, hearing, speech, dysphagia
**Grade 4**	Bilateral ptosis
**Grade 5**	Severe dyspnea
**Grade 6**	Severe consciousness disorders, motor and respiratory paralysis

Antivipmyn® Africa, manufactured by Bioclon Institute (Mexico), is composed of highly purified lyophilized F(ab’)_2_ immunoglobulin fragments [11]. It is produced by immunization of horses with the venoms of *Bitis gabonica*, *B. arietans*, *Echis ocellatus*, *E. leucogaster*, *E. pyramidum*, *Naje haje*, *N. melanoleuca*, *N. nigricollis*, *N. pallida, Dendroaspis viridis* and *D. polylepis*. Preclinical testing indicated a specific neutralizing potency of more than 250 LD_50_ per vial against all relevant species [11]. Administration of the antivenom was always by direct intravenous push as detailed elsewhere and modified as indicated in the Results section [9].

Statistical analysis used Wilcoxon rank sum test for time to presentation, score and time to death, and Student’s *t* test for treatment doses, with p = 0.05.

## Results and discussion

In the region of Upper Guinea, we analyzed 226 records of patients bitten between 2005 and 2006. At the Pasteur Institute of Guinea, in Lower Guinea, 521 patients were treated from 2009 to 2011.

**Figure 2:**
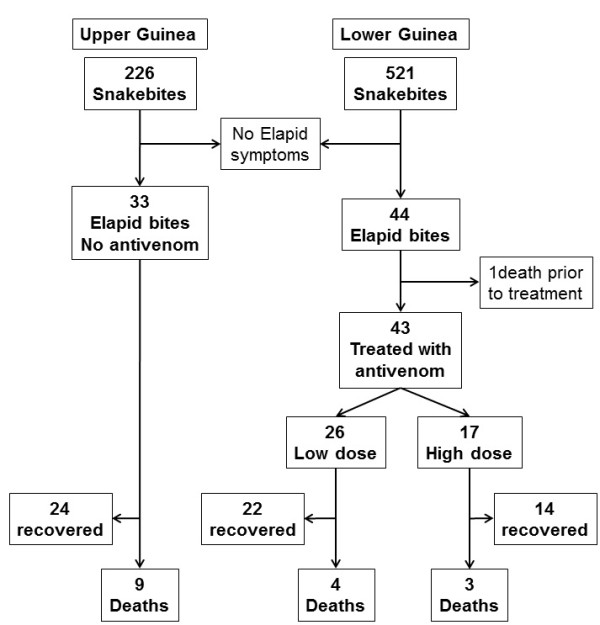
Flow chart of inclusion and outcome of patients from Upper and Lower Guinea.

The data collected as well as the outcomes are summarized in Figure 2 and Tables 2, 3, 4, 5. There were no significant differences either in the neurological scores on arrival or in the delay of treatment between the three groups. Administration of antivenom, independently of dose, did not significantly reduce CFR between the untreated group from Upper Guinea and either group treated in Lower Guinea.

**Table 2:** Symptoms and time to death in patients of group 1

**N**	**Time of the bite**	**Sex and age**	**Treatment**	**Symptoms at presentation**	**Time of the death**	**Time between bite and death**
1	8:10 a.m.	M – 15	Traditional	Hypersalivation; blurred vision	10:45 a.m.	2:35
2	9:00 p.m.	M – 43	Symptomatic	Breathlessness	4:00 a.m.	7:00
3	3:20 p.m.	F – 56	Traditional and symptomatic	Weakness; sweat	11:10 p.m.	7:50
4	11:00 a.m.	M – 23	Symptomatic	Ptosis	7:00 p.m.	8:00
5	0:50 p.m.	M – 17	Traditional	Blurred vision	2:15 p.m.	1:25
6	4:20 p.m.	F – 32	Traditional and symptomatic	Consciousness disorders	1:00 p.m.	20:40
7	8:00 p.m.	F – 8	Symptomatic	Blurred vision and consciousness disorders	12:30 a.m.	16:30
8	5:00 p.m.	F – 36	Traditional	Hypersalivation, consciousness disorders	4:10 a.m.	11:10
9	2:30 p.m.	M – 51	Traditional and symptomatic	Local bleeding; breathlessness	Day 2: 6:00 p.m.	27:30

**Table 3:** Symptoms and time to death in patients of group 2 (low dose of antivenom)

**Patient**	**Time between bite and presentation**	**Clinical score on arrival**	**Snake species**	**Vials of antivenom**	**Associated symptoms**	**Outcome**
8	3	4	*D. viridis*	2		Recovery H_6_
16	21	5	*D. viridis*	4		Recovery H_6_
17	11	3		1		Recovery H_6_
19	18	6	*D. polylepis*	4	Edema	Death H_7_
20	48	2		1		Recovery H_3_
26	5	5	*D. viridis*	4		Recovery H_6_
27	6	6		4	Local bleeding	Recovery H_6_
29	2	6		2	Local bleeding	Death H_1_
33	47	2		1		Recovery H_3_
37	2	1		2		Recovery H_3_
38	13	3		1	Edema	Recovery H_3_
53	15	1		1	Local bleeding and edema	Recovery H_3_
59	4	4		2	Bleeding	Recovery H_3_
71	10	4		4	Edema	Recovery H_6_
77	20	3	*D. viridis*	2	Edema	Recovery H_3_
87	7	5	*D. viridis*	6		Recovery H_48_
88	4	5		4		Recovery H_24_
119	3	5	*D. viridis*	2		Recovery H_24_
123	3	4		1		Recovery H_6_
124	10	5		2		Recovery H_24_
130	3	1		2	Local bleeding	Recovery H_6_
134	14	3	*Naja* sp.	4	Edema	Death H_4_
138	4	2		3		Recovery H_6_
141	2	2	*D. polylepis*	6		Death H_7_
148	6	4	*Naja* sp.	2	Edema	Recovery H_12_
149	1	2	*N. melanoleuca*	1	Local bleeding	Recovery H_6_

H: hour after initial antivenom dose.

**Table 4:** Symptoms and time to death in patients of group 3 (high dose of antivenom); one death (#11) was not recorded because the patient died before treatment, less than three hours after the bite

**Patient**	**Time between bite and presentation**	**Clinical score on arrival**	**Snake species**	**Vials of antivenom**	**Associated symptoms**	**Evolution**
1	2	2		4		Death H_2_
2	7	4	*D. viridis*	4	Edema	Recovery H_31_
3	7	4	*Naja* sp.	4	Edema	Recovery H_79_
4	2	1		4		Recovery H_50_
5	3	2	*Naja* sp.	4		Recovery H_51_
6	3	5	*D. polylepis*	4	Local bleeding	Death H_1_
7	1	1	*N. nigricollis*	4	Edema	Recovery H_49_
8	2	4		4	Local bleeding	Death H_2_
9	4	1		4	Local bleeding	Recovery H_28_
10	1	2		4		Recovery H_49_
12	4	4	*Naja* sp.	4	Edema	Recovery H_52_
13	4	1		4		Recovery H_28_
14	5	6		4	Edema	Recovery H_29_
15	6	4	*D. viridis*	4		Recovery H_30_
16	5	3		4		Recovery H_53_
17	10	6		6		Recovery H_34_
18	18	2		4		Recovery H_66_

H: hour after initial antivenom dose.

**Table 5:** Comparison of score and time to death in the 3 groups

	**Group 1**	**Group 2**	**Group 3**
	**(no antivenom)**	**(low dose)**	**(high dose)**
**Number of cases**	33	26	17
**Score at presentation**	Cured patients = ?	Cured patients = 4 [2–5]	Cured patients = 2.5 [1.3-4]
Median [Q:0.25-0.75]	Fatalities = 5 [3–6]*	Fatalities = 4.5 [2.8-6]	Fatalities = 4 [3–4.5]
**Mean of antivenom** ± SD [95%CI]	0	26.2 ± 5.9 mL	41.2 ± 2.3 mL
**Number of fatalities**	9 (27.3%)	4 (15.4%)	3 (17.6%)
**Time to presentation**	–	Cured patients = 5 [3.3-9.3]	Cured patients = 3 [3.3-10]
Median [Q: 0.25-0.75]		Fatalities = 2.5 [2–6.8]	Fatalities = 2 [2–8]
**Time to death**	8 [7–13.5]	5.5 [3.3-7]	4 [4–4]
Median [Q:0.25-0.75]			

*Score established retrospectively from recorded clinical symptoms.

Data collection varied among the three groups due to the conditions of the surveys and the treatment protocols. The study in Upper Guinea was retrospective, a fact that restricted the available information. In the two groups from Lower Guinea, the treatment protocol changed due to the apparently low efficacy of the Antivipmyn® Africa antivenom, as well as the modalities of patient surveillance and the recovery criteria, which introduced some intergroup heterogeneity. Some comparisons could not be made among all groups, such as the delay in receiving a consultation (this variable was not available for the untreated group) or the time between bite and recovery in surviving patients (since none of the groups followed the same recovery criteria). In addition, Elapid species differ slightly in these two aspects (Table 6). In the end, the only parameters usable for comparison were the score on arrival, the dose of antivenom, case fatality rate and the time elapsed between bite and death (Table 6). As a consequence, comparability between groups, as well as patient series described in the literature, is limited. It appears, however, that treatment failures are common even when employing high doses of antivenom associated with intensive symptomatic treatment (mechanical ventilation, prostigmine etc.).

**Table 6:** Elapid species and distribution in Upper and Lower Guinea

**Species**	**Upper Guinea**	**Lower Guinea**	**Symptoms**
***Naja nigricollis***	Common	Common	Local necrosis, neurotoxic signs
***Naja katiensis***	Common	Absent	Neurotoxic signs, local necrosis
***Naja melanoleuca***	Common	Common	Neurotoxic signs
***Dendroaspis viridis***	Present	Common	Neurotoxic signs, muscarinic syndrome
***Dendroaspis polylepis***	Present	Present	Neurotoxic signs, muscarinic syndrome
***Pseudohaje nigra***	Absent	Rare	No envenomation recorded

**Figure 3:**
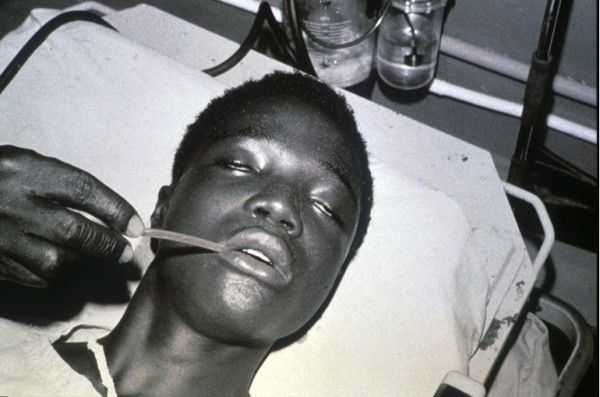
**Neurotoxic syndrome due to *Naja melanoleuca *bite (note pathognomonic ptosis) Photo by E. Stahel.**

**Figure 4:**
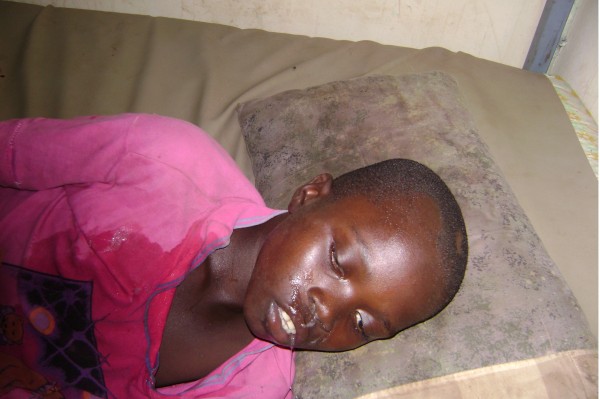
**Associated neurotoxic and muscarinic syndromes due to *Dendroaspis *sp bite (note hypersalivation and profuse sweating) Photo by C. Baldé.**

Our analysis of the records from the two health centers allowed us to identify 77 patients who, on arrival, presented neurological troubles strongly suggestive of envenomation by Elapidae. In some cases species could be identified either by examination of the specimen or by a description. Furthermore, the observed symptoms permitted, with a little experience, educated guesses of the species or at least genera responsible for the envenomation (Table 6). Some species of *Naja* are responsible for an isolated neurological syndrome (*syndrome cobraïque*) (Figure 3), or associated with local necrosis in the case of envenomations by *Naja nigricollis* and *N. katiensis* (both spitting cobras). *Dendroaspis* (in Guinea, *D. viridis* and *D. polylepis*) envenomation is also associated with the syndrome, a muscarinic symptomatology manifested by abundant sweat, sialorrhea, vomit, diarrhea and mydriasis [12] (Figure 4). We do not have information on envenomations by *Pseudohaje nigra*.

A delay until consultation, which is detailed only for patients in groups 2 and 3, appears to be a decisive factor insofar as it delays treatment, which is more effective the earlier it is administered [13-15]. The lack of significance between the groups could result from the small number of patients. Paradoxically, the delay in consultation is longer (although not significantly) in patients who recovered in comparison to those who died (Table 5). However, some severely envenomed patient could have died before reaching the health center. But there is no evidence to support this hypothesis. In addition, the time between bite and fatality in group 3 is remarkably shorter and significantly different from that of the other two groups. The duration of hospitalization is also much shorter, which suggests either that envenomations were more severe (even if scores on arrival were similar) or that shorter delays were declared.

The efficacy of Antivipmyn® Africa in these cases is therefore directly questioned. There is no reason to doubt its experimental neutralization capacity, but these results do not seem to be transposed to the clinical situation [11]. Dosage does not seem to be the issue, as the same results are apparent at lower and higher doses. In our study, doubling the initial dose did not lead to a difference in the maximal dose administered to the patients (60 mL), a fact that suggests that the criteria for re-administration of antivenom were not apparent during the monitoring of the patients. But despite demonstrable neutralization in the animal model, it is possible that the venom of a particular species (of a total of four possible ones in Lower Guinea) is poorly neutralized by the antivenom in humans; given the small sample of patients and the lack of unambiguous snake identification in most of them, this could skew the overall results and mask some measure of overall efficacy. It is also possible that the therapeutic window is simply very narrow for some or all venoms.

More generally in Elapid envenomations, such low apparent clinical efficacy has also been observed by numerous authors who have obtained similar results, even at much higher doses (Table 7). Furthermore, in all other studies, antivenom use was associated with mechanical ventilation on demand, as well as other symptomatic treatments such as cholinesterase inhibitors. These were not available for patients in the present study. Although the species, and therefore the toxicity and mode of action of their venoms, are different, and the antivenoms – whose neutralizing capacities are also variable – are not the same, it is important to note that the treatment of Elapidae envenomations is less effective than treatment of those inflicted by Viperidae. In fact, the doses reported by numerous authors (surpassing occasionally even a hundred vials, a tendency which confirms a low level of efficacy) seem unreasonable, with regard to both the quantity of heterologous protein administered and the cost of treatment, already prohibitive for extremely poor patients even at much lower doses.

**Table 7:** Antivenom doses and mortality observed in the literature

**Countries**	**Patients (n)**	**Mean doses (mL [range])**	**# deaths (CFR)**	Supportive measures*	**Reference**
Guinea	26	25 ml	4 (15%)	No	Group 2 this study
		[10–60]			
Guinea	17	40 mL	3 (18%)	No	Group 3 this study
		[40–60]			
India	28	150 mL	0	VS	[16]
India	27	600 mL	3 (11%)	VS	[16]
		[300–1.600]			
Sri Lanka	87	> 100 mL	5 (6%)	VS	[17]
		[100–200]			
Sri Lanka	25	100 mL	2 (8%)^§^	VS	[18]
		[80–200]			
India	37	150 mL	11 (22%)^*^	VS; AC	[19]
Taiwan	22	30 mL	0	VS	[20]
		[10–100]			
Papua New Guinea	139	50 mL	6 (4.3%)	VS	[13]
Papua New Guinea	31	25 mL	0	VS; AC	[13]
India	14	60 mL	1 (7%)	VS	[21]
India	12	120 mL	0	VS	[21]
Thailand	68	100 mL	0	VS	[22]
India	86	510 mL	3 (3.5%)	VS	[23]
		[50–1900]			
Papua New Guinea	156	50 mL	3 (1.9%)	VS	[14]
Vietnam	42	50 mL	0	VS	[24]
		[50–100]			
Thailand	85	40 mL	1 (1%)	VS	[25]
		[10–200]			

*VS*: ventilatory support; *AC*: anticholinesterase; ^§^the patients died from extensive necrosis; ^*^three patients died before treatment.

In hemorrhagic envenomations in the West African savanna, a rapid arrest of hemorrhage and coagulopathy was apparent after antivenom administration, requiring no additional care to remove most patients from immediate danger of life-threatening hemorrhage [7-9]. The lack of therapeutic response in neurotoxic envenomations, however, suggests that antivenom alone may not suffice to ensure a sufficiently rapid recovery to prevent respiratory failure and death. Under the extremely precarious conditions of this study, potential benefits of antivenom administration, such as a speedier recovery from respiratory paralysis, would be masked by the unavailability of assisted ventilation.

One possibility is that the antivenom has a low efficacy after neurotoxins are fixed on the neuromuscular receptors, which usually happens in the first few hours after the bite [26]. In this view, the problem is immunochemical: those epitopes recognized by the antibodies would be masked on receptor-bound toxins and this would in turn prevent toxin-antibody complex formation, neutralization and elimination.

A second hypothesis that merits attention is pharmacokinetic: the toxin-antibody encounter does not occur because the antigen and the antibody are not found in the same biological compartment. The antivenom is largely present in the vascular compartment [27]. It is in this compartment that antigen-antibody complex formation occurs, provided that the relevant venom components are there as well. It has been clearly shown that Viperidae venoms are found in the blood, where they are bound by antivenom [28]. To the best of our knowledge, it has never been shown that neurotoxins from Elapidae venoms were present in high proportion in the vascular compartment, nor that neurotoxins are bound by F(ab')_2_ after intravenous administration.

## Conclusion

During a clinical study in Guinea under true field conditions, the administration of purified immunoglobulin fragments to treat neurotoxic enevenomation due to Elapidae has proven a disappointment, in agreement with the results of numerous other clinical studies.

The hypothesis of insufficient venom neutralization is difficult to maintain because, on one hand, the experimental neutralization of venoms is generally acceptable and, on the other hand, this divergence has been noted for many elapid venoms and antivenoms throughout the world. Alternatively, a hypothesis of an absence of toxin-antibody complex formation merits consideration; it could come about by epitope masking on receptor-bound toxins and/or by a failure of the antibodies to encounter the relevant toxins due to pharmacokinetic constraints. In the first case, the only recourse would be very early administration of antivenom or the development of neutralizing antibodies against epitopes not masked on receptor-bound toxins. In the second case, another route of antivenom administration should be considered.

In the meantime, it is advisable to administer antivenom as early as possible after the bite to attempt to eliminate available venom antigens and to secure assisted ventilation in case the patient presents the onset of respiratory distress.

## Consent

Written informed consent was obtained from the patients for publication of this study and any accompanying images.

## Competing interests

The authors declare that there are no conflicts of interest.

## Authors’ contributions

JPC, RPS and AM designed the study. MCB, JPC and AM wrote the protocol. MCB and MYB performed the field study. MCB, JPC analysed the results. JPC wrote the draft. All authors corrected and validated the paper. MCB and JPC are garantors of the paper.
